# Patients’ Experiences With Complex Regional Pain Syndrome (CRPS) and a Rehabilitation Program That Combines Graded Motor Imagery (GMI) and Tactile Desensitization (TD)

**DOI:** 10.1155/prm/4480549

**Published:** 2026-04-02

**Authors:** Isak Furu Krogstad, Lena Danielsson, Louisa Palmi Danielsson, Gunnvald Kvarstein, Svein Bergvik

**Affiliations:** ^1^ Department of Psychology, The Arctic University of Norway, Tromsø, Norway, uit.no; ^2^ Anesthesia and Critical Care Research Group, Department of Clinical Medicine, The Arctic University of Norway, Tromsø, Norway, uit.no; ^3^ Pain Department, Division of Surgery, University Hospital of North Norway, Tromsø, Norway, unn.no

**Keywords:** adherence, complex regional pain syndrome, graded motor imagery, illness perception, pain, patient experiences, qualitative

## Abstract

Complex regional pain syndrome (CRPS) is characterized by predominant pain in combination with sensory, trophic, motor, or autonomic changes. Comprehensive rehabilitation programs have demonstrated promising results, but the treatment protocols can be challenging to apply within clinical settings. Intensive rehabilitation programs require highly motivated and dedicated patients, and insight into patients’ perspectives and experiences of the treatment is crucial for ensuring adherence and preventing drop‐out. To our knowledge, no previous qualitative studies have explored the challenges underlying a comprehensive home rehabilitation program for patients with CRPS. This study presents a qualitative study of ten CRPS patients who received or had recently completed an individualized Graded Motor Imagery (GMI) and Tactile Desensitization (TD) treatment. Semistructured interviews were conducted using a phenomenological approach. The analysis followed a four‐step process based on systematic text condensation (STC). Illness perception emerged as an overarching theme, and how patients perceived and understood their condition and the treatment appeared to impact the treatment and its outcome significantly. Four subthemes emerged: (1) illness comprehension; (2) regaining control; (3) altered perceptions of the affected limb; and (4) provider support. The participants described how their perception and comprehension of the condition and symptoms changed during treatment. Illness comprehension, regaining control, reintegrating the affected limb, and strong clinician support seemed vital to the positive change. These factors may help future clinicians implement a home‐based treatment program for CRPS patients and increase the likelihood of adherence and successful treatment outcomes.

**Trial Registration:** ClinicalTrials.gov identifier: NCT02753335

## 1. Introduction

Complex regional pain syndrome (CRPS) is a multifactorial condition characterized by a dominating symptom of pain combined with sensory, trophic, motor, or autonomic changes [1, 2]. Symptoms may also include body perception disturbances (BPD), body image distortions, and various motor dysfunctions of the affected limb [3]. The condition can be persistent and debilitating, causing high levels of suffering and disability over time [4]. Incidence rates vary, ranging from 5.5 to 26.2 cases per 100,000 person/year, and are 2.5 to 4.3 higher among women than men [5–7].

Fractures, tissue damage, surgical traumas, and immobilization are known triggers of CRPS [1, 8]. Several potential pathophysiological mechanisms of CRPS have been described, including autonomic dysfunction, autoimmune involvement, exaggerated inflammation, genetic factors, and maladaptive neuroplasticity [2, 9, 10], but the interactions and contributions of these mechanisms remain incompletely understood [1].

Various treatment modalities have been introduced, including physical therapy, rehabilitation approaches, neurostimulation, pharmacological treatment, and surgical interventions [1]. Although there is little high‐quality evidence for current treatments [11], there is strong consensus for an individualized, interdisciplinary approach that incorporates education, pain relief, physical rehabilitation, and psychological intervention [12].

Graded Motor Imagery (GMI) is a rehabilitative intervention for various chronic pain conditions, such as postamputation limb pain, back pain, and CRPS [13, 14]. The aim is to gradually activate sensory and motor cortical areas through systematic left/right discrimination training, imagined hand movements, and mirror therapy [13].

GMI shows promising effects on pain and function [11, 14, 15], although the scientific quality of the evidence is low [11]. A small‐scale multicenter study reported improvements in physical function and left/right discrimination performance but no effect on pain intensity [16]. The disappointing results were partly explained by low adherence to the intensive treatment protocol and an underpowered study [16].

Tactile Desensitization (TD) [17, 18] aims to reduce hyperalgesia, allodynia, and neglect through repeated, gradually increased stimulation of the affected area with various materials [17, 19, 20]. Correcting the incongruency between motor intention and sensory feedback may reduce pain and sensory changes [21]. TD is recommended as part of a multimodal treatment approach for CRPS [22].

The comprehensive GMI treatment protocol may be challenging to apply in clinical settings [15, 16], both for patients and healthcare providers, and combining TD may further complicate this. Thus, understanding the potential barriers and facilitators affecting patients’ adherence to a comprehensive home treatment program is crucial to achieving successful treatment outcomes [15, 16].

According to the Common Sense Model of self‐regulation, patients’ self‐management and coping with illness are guided by their illness representations [23], with established effects on outcomes [24]. In CRPS, negative illness perceptions indicating a poor understanding of the condition, are associated with higher levels of pain, kinesiophobia, and disability [25]. Clearly, understanding patients’ perspectives and experiences is critical for successfully designing interventions, providing necessary support and follow‐up, and ensuring adherence.

To our knowledge, no qualitative studies have explored the experiences of individuals living with CRPS who undergo an individualized home rehabilitation treatment combining GMI and TD.

This study aimed to investigate the illness and treatment experiences of patients with CRPS who were currently or had recently received an individualized GMI and TD.

## 2. Methods

We conducted an interview study using a phenomenological approach to explore how patients experienced their illness and treatment [26, 27]. The study was part of the research project “A Randomized Trial of Patients With Complex Regional Pain Syndrome Comparing Graded Motor Imagery and Desensitization Versus Simple Desensitization and Changes in Resting‐State Connectivity of Cerebral Networks Before and After Treatment.”

### 2.1. Procedure

Participants were recruited among patients with a potential CRPS referred to the Pain Department at the University Hospital of Northern Norway (UNN). Those with a confirmed CRPS according to the Budapest criteria recommended by the International Association of Pain (IASP) [22, 28] were provided information about the treatment and the study their motivation to participate was determined, and their tolerance to the GMI and TD procedures was tested. None of the participants were undertaking other treatments during the procedure. Participants were recruited to the interview study via purposeful sampling [29] among patients with a confirmed CRPS diagnosis [22, 28] who were currently receiving or had recently completed a GMI and TD rehabilitation program within the past 12 months.

Individual, semistructured, in‐depth interviews were performed following Kvale and Brinkmann’s description [27], using open‐ended questions and a prepared interview guide as a starting point. The interview guide included questions on the origin of their pain and symptoms, their understanding of the illness, how it affected their lives, and their experiences with the rehabilitation program. Several additional topics were covered during the interview. A pilot interview was conducted to strengthen the quality and validity of the interview guide and method [27, 30]. The participants chose a convenient location for their interviews, either at their homes or in a private session room at the university. The interviews were conducted by the first author (IFK), a trained medical doctor with some experience treating patients with CRPS. The interviews varied in length from 1 to 2.5 h. Memos were taken during and after each interview to preserve thoughts and ideas from the session.

### 2.2. GMI and TD

The current rehabilitation program is an adaptation of GMI and TD [31] provided by the Pain Department at the University Hospital of Northern Norway (UNN), administered by a senior clinician with expertise in chronic pain management. Patients received comprehensive information about CRPS and the potential impact of coexisting psychosocial factors on pain. When needed, they were offered support and guidance to help them manage these challenges.

The program comprises a series of treatment sessions, including the GMI techniques of left/right discrimination training, motor imagery exercises, mirror therapy, and TD. In the left/right discrimination task, patients are asked to determine whether the photographs of the limb in various positions depict a left or right limb [32]. During the motor imagery task, patients are instructed to imagine the movements of the affected limb. In mirror therapy, the affected limb is hidden, and the patient observes the mirror image of the unaffected limb. This creates a visual impression of the affected limb being pain‐free and functional. Two different TD techniques are applied. The first technique begins by testing both sides, comparing the affected to the unaffected side. The next step involves gradually stimulating the skin in the affected area using five different materials. This may feel unpleasant, and if the pain worsens 24 h later, the stimulus intensity is adjusted. Each treatment is individually tailored to the level of unpleasantness and/or pain, as well as the patient’s progress and response to the treatment. In the second TD technique, the patient uses the affected hand or foot to search for an object hidden in one of three boxes filled with rice, puffed rice, or beans, respectively. Initially, the object is typically large and easily identifiable. As the patient progresses, the difficulty increases as the objects become smaller and more similar to the contents of the box.

The treatment program progresses through monitored steps, gradually becoming more difficult as progress is made. The process is continuously evaluated and adjusted [33]. Due to individual differences in tolerability to the treatment steps, the intervention is individually tailored. Based on our experience, the treatment requires a flexible approach that adapts to each patient rather than a manual‐based procedure with fixed instructions on frequency, duration, and the progression of treatment steps.

The patients are instructed to practice at home in multiple sessions per day. While consultations are initially arranged once a week, time intervals gradually increase during the program. A typical treatment course may span 6–12 months, although there is individual variation.

### 2.3. Analysis

Interviews were audio‐recorded and transcribed by IFK. All information that could directly or indirectly identify the participants was deleted or altered, and names were replaced with pseudonyms to ensure anonymity. Manual data analysis was combined with NVivo12 software for storage and overview [34]. The analysis followed the systematic text condensation (STC) approach described by Malterud [35]. The method involves the following steps: (1) repeated listening to the audio recordings and reading through the transcripts to form an overall impression and overview; (2) identifying meaningful units; (3) abstracting the content of the meaning units into condensates; and (4) putting it all together and identifying the meaning behind the content. The themes and meaningful units were not preconceived before the interviews but emerged from reading and interpreting the data. In the first steps, the interview transcripts were read and preliminarily analyzed independently by three co‐authors (I. F. K., L. P. D., and S. B.). The following steps were a joint analytical effort by the group of authors. In a series of meetings, meaningful units and key themes from each researcher’s reading of the transcripts were discussed and compared to strengthen the analysis [36]. The themes were chosen based on their richness and essence rather than frequency [35].

### 2.4. Ethical Considerations

All participants received oral and written information about the study and signed a written consent form in accordance with the Regional Committee for Medical and Health Research Ethics (REK) requirements. Approvals were obtained from the Data Protection Officer at the University Hospital of North Norway, and from REK (Reference number 2014/1671‐REK Nord).

## 3. Results

Ten participants (one male and nine females), aged 23–73, completed the interviews (see Table 1). All participants were white Caucasian. The pilot interview was deemed valuable for inclusion, as it enriched the dataset.

**TABLE 1:** Demographics.

Pseudonym	Age	Gender	Affected limb (s)	Duration of CRPS
# 1 Tracy	51	Woman	Left upper limb	2‐3 years
# 2 Claire	55	Woman	Left upper and lower limbs	1 year
# 3 Elise	40	Woman	Left lower limb	2 years
# 4 Johanna	73	Woman	Right upper limb	2 years
# 5 Jenny	23	Woman	Right upper limb	7 years
# 6 Laura	37	Woman	Right lower limb	6 years
# 7 Thomas	70	Man	Right upper limb	1 year
# 8 Joan	53	Woman	Left upper and lower limbs	2 years
# 9 Ada	62	Woman	Left and right upper limbs	10 years
# 10 Riley	65	Woman	Right upper limb	6 months

The participants gave detailed descriptions of how they perceived their condition, how it influenced their daily life, their efforts to cope with it, their experience of the treatment they received, and how it affected their symptoms and altered their illness perception and self‐efficacy.An overarching theme across the interviews was illness perception. How participants perceived and understood their condition and the treatment seemed to significantly impact their adherence to the treatment and its outcome. Within the core theme of illness perception, four subthemes emerged: (1) illness comprehension; (2) regaining control; (3) altered perceptions of the affected limb; and (4) provider support. Each theme played a crucial role in the participants’ lives.

### 3.1. Illness Comprehension

Illness comprehension encompasses various factors, including past illness experiences with CRPS, understanding of the condition, and integrating new information into everyday life. CRPS was perceived as a complex and intricate condition with frightening and frustrating symptoms.

“Before I received the diagnosis, I kept wondering: where does this pain come from? That’s the thing … when you have a big wound on your leg, you know that it is the wound on your leg that hurts. But when you can’t see anything. All there is … unbearable pain.” (Ada)

“*I had excruciating pain and swelling on my arm (the affected limb). When I received the diagnosis, I understood that there was a reason for it. Having a name for it and somewhere to place it. It was a sense of relief there*.” *(Riley)*

All participants stated that the information provided by the clinician helped them understand the nature of the illness and the rationale for the treatment. They reported a significant change in how they perceived and understood the condition. Insight into the condition and pain mechanisms helped them adhere to the treatment program and improve coping with the CRPS. Finding an explanation was essential. Receiving a diagnosis seemed to promote acceptance of the condition, including its various symptoms and how it affected them. This shift is exemplified in Joan’s experience:

*“I think that without learning about this syndrome, I would’ve become a lot worse. … I was in so much pain that my husband almost couldn’t touch that side. I became increasingly careful with the affected limb because we had no idea why this was happening. I think I would’ve become more and more careful about the whole side. I would’ve become stiffer and stiffer, more and more immobilized.” (Joan)*

*“When she (the doctor) started telling me and explaining how this worked, once I began to understand how pain worked, I understood why my hand (the affected limb) always tries to bend and rotate inwards when I am in pain. Oh my god, what a relief! I can’t explain the level of relief! I am not crazy!” (Joan)*

Some emphasized the importance of recognizing that pain itself was not dangerous and how this insight helped them gain more control of their condition and life.

*“Receiving a diagnosis and understanding more of what this is … now I know that pain is not dangerous. I can use my arm, and it will not make it worse. Someone can touch me, and it won’t break me. I feel much safer now. I know that it is not dangerous.” (Jenny)*

*“It is not dangerous. You are not going to die from it. You must stand up and fight it. It is a war you face every day. But how you face it, that is up to you.” (Joan)*

Several participants admitted that before meeting the clinician specialized in CRPS, they had hoped for a cure, a “quick fix” that would resolve their CRPS condition, perhaps in the form of a pill. Receiving information about CPRS and the involved mechanisms helped them understand their symptoms, what was going on in their bodies, what to expect from the condition, and the rationale for the intensive training and treatment. This newfound understanding enhanced their motivation and commitment to follow the demanding program.

“*I was told that this will not be easy. There are no pills for this. Training is what it takes to get better. Okay. Once I understood that, I took it from there.” (Ada)*

*«The doctor explained to me that this is going to take time. Not a week, not a month, but time. After I realized that … everything started to run more smoothly.” (Riley)*

However, not everyone shared this perspective of pain. One participant maintained a belief that pain was something out of one’s control.

*“The pain … it is not going to change … it is just there. It is like tinnitus! You try and push it away.” (Thomas)*

*“The more you think about it (the pain), the worse it gets.” (Thomas)*

The participants expressed in various ways how the information provided by the clinician and their experiences during training enhanced their comprehension of CRPS and the pain. Living in a state of uncertainty without any diagnosis or knowledge about what was going on in their body had been highly disruptive. The first major step was receiving diagnostic information. Knowledge of the mechanisms and current theories of the condition helped shape their understanding of CRPS, how pain works, and develop realistic treatment expectations. Expectations involved how the condition might affect their lives and what to anticipate from the treatment. All these factors helped form and guide their understanding of the illness. The participants’ experiences indicated that tailoring the therapy to their comprehension and current state was essential for optimizing the treatment and improving outcomes.

### 3.2. Regaining Control

CRPS was experienced as a challenging and disabling condition that turned their lives upside down. It led to a sense of losing control over their body and disrupted the balance in their daily life.

*“I couldn’t walk like I used to, I couldn’t run like I used to, I peed myself, I shat myself … and then with my left side not working (affected side). It was all chaos.” (Joan)*

“*He (the affected limb) runs my life*. *I don*’*t run his.” (Laura)*

In Laura’s quote above, the condition itself was personified as *he*, which illustrates its massive impact on her life. Other participants described losing their ability to do activities they loved, such as hiking, training, or even simple everyday household tasks or gardening. Several faced difficulties in being intimate with their partner. They described in various ways how the treatment program and consultations initiated a process of regaining control. For some, this meant focusing on the treatment program, and they emphasized that the early positive effects of the training motivated them to continue with the exercises. For others, it involved finding their own way and regaining independence.

*“This actually works (the exercises)! Once I felt it worked, it became easier. I started to believe in it.” (Tracy)*

*“I did it every day (the program). Had a regular routine. It helped to have a plan and regulate up and down. I could feel the exercises helping. That became a motivator in itself. You felt like you had regained control.” (Riley)*

Taking back control emerged as a key theme for regaining a sense of agency over their bodies and lives. Others described control as a choice, as an act toward mastering the condition and their situation:

*“This won’t stop me, I am going to be the master of my disease.” (Ada)*

Taking back control of their lives proved to be a great challenge for all the participants. CRPS had a massive impact on their lives and abilities. Many of the participants described in various ways a journey from being immobilized and without control toward taking back their lives through gaining ownership of the treatment. However, this was not the case for all of them. Some did not describe any process of regaining control and still felt that external factors decided how their condition developed, which greatly influenced how they lived. Control and a sense of agency seemed to play an important role in the participant’s ability to adhere to the treatment and regain ownership of their own lives.

### 3.3. Altered Perceptions of the Affected Limb

Another important theme was how the CRPS condition disturbed their perceptions of their body and the affected limb(s).

*“In the beginning, it wasn’t my hand. It was just something that hung there. Something I had to be careful with. It felt big and clumpy.” (Clare)*

*“I rejected it (the affected limb), you know? But, no, it was not there. It was not mine. It was not a part of me. It wasn’t a part of my whole.” (Joan)*

All participants described how their symptoms led to changes in their behavior and how they used their affected limb. Altered body perceptions seemed to increase their challenges and hinder improvements in function and recovery from CRPS. One of the participants disregarded the affected limb for weeks before recognizing it with the help of her clinician. Becoming aware of these symptoms and their negative implications had been an important topic in their consultations with the clinician, and this awareness became a motivator for them to actively incorporate the affected limb into the treatment and back into daily activities.

*“You might as well have chopped off the arm (the affected limb) … I couldn’t use it for anything. It hurt, it was swollen … it was just in the way! That was the feeling. But then I started using it more and more. Gradually, it started becoming a part of me again. I could start using it again … it was a process. It has taken a long time.” (Tracy)*

All participants found it difficult to progressively reintroduce the affected limb into everyday life. Becoming aware of and consciously using it as much as possible took a lot of effort, but it was also highly rewarding. Many participants described it as a gradual process of familiarizing themselves with all aspects of life until it once again became an integrated part of themselves.

“*When I started using the hand again (affected limb), I was living a new life.” (Johanna)*

*“I decided I had to incorporate the hand (affected limb). You have to be a part of the team. Then it started to get better.” (Ada)*

Ada’s quote illustrates an interesting example of altered perceptions of the affected limb. She used “you” to refer to the affected limb, which she regarded as separate from herself. At times, it would feel lifeless, while at other times, there would be excruciating pain. She started doing exercises daily, directing her attention toward the hand, and gradually incorporated it into her life.

Altered perceptions of the affected limb were reported by all the participants to varying degrees. It can be challenging for the participant and the clinician to discover how these disturbed perceptions present themselves. These symptoms were barriers to adhering to the treatment and improving the CRPS. Thus, for some participants, these disturbed body perceptions were among the critical issues in their consultations with the clinician.

### 3.4. Provider Support

The participants considered having a supportive and dedicated clinician crucial to the treatment. All participants were followed closely by the same medical doctor (pseudonym: Doctor J) throughout their treatments. They deeply valued supportive counseling and having someone to turn to when facing difficulties.

*“To be taken care of by someone. That eases the pain a bit. Just by getting help. Someone who listens to you, who sees you and a shoulder to cry on. It helps.” (Ada)*

*“These consultations with Doctor J were crucial. It goes up and down, and sometimes I feel like I can’t do this. I just can’t … I had a lot of focus on what I couldn’t do. But Doctor J supported me, and that support was vital.” (Riley)*

The clinician’s support over time meant a lot for the participants. It helped them maintain their motivation and spirits and improved their adherence to the intensive treatment program. This reflects the considerable power and influence patients attribute to a supportive clinician. The interviews indicate that their clinician used this privileged position to guide and assist the patients in adhering to their treatment.

*“To have that support. That encouragement. You need it.” (Clare)*

One participant described a pause in the follow‐up from the clinician for a period due to some unexpected circumstances. He explained how this had a detrimental effect on his adherence to the treatment:

*“It was demoralizing. Not having that support. I had to use so much more energy to try and get the exercises done.” (Thomas)*

Another issue among the participants was the ignorance they had encountered among physicians and healthcare professionals regarding CRPS. Some felt they were not believed and were told that “it is all in your head,” which increased their feelings of loneliness and isolation. The lack of validation can damage the clinician–patient alliance and negatively influence treatment.

## 4. Discussion

This qualitative study explored how patients living with CRPS experienced a tailored GMI and TD rehabilitation program. The findings provided valuable insight into the challenges they face when adhering to a demanding, home‐based self‐management treatment for CRPS, such as this combined GMI and TD program.

Illness perception emerged as the central theme, including four subthemes. These themes partly align with the five dimensions of the Common‐Sense Model (CSM) of patients’ illness representations: identity, consequences, cause, timeline, and cure/control [23, 37]. Knowledge of the illness helps the patient gain control over one’s situation and life [38, 39]. In general, a patient’s illness comprehension may differ greatly from the view held by their clinician [40, 41], which may influence adherence and the treatment outcome [38, 41, 42].

The first major theme, *illness comprehension*, closely resembles the identity, cause, and timeline dimensions of the CSM. This finding corroborates earlier findings [25, 43]. Thorough and tailored information provision can help the patient form a unified view of the nature, causes, expected timeline, and duration of the illness. It is well known that divergent interpretations of the illness can damage the patient–clinician alliance and hinder further collaboration and adherence [40, 44]. Several participants expressed a shift in their expectations regarding the CRPS, from hoping for a “quick fix” to acknowledging that it will take time. Their clinician had emphasized this during treatment, and it helped them avoid unrealistic expectations of the treatment. That was crucial for their adherence to the treatment over time.

The clinician needs extensive knowledge to explain complex conditions such as CRPS effectively, capture the patient’s understanding, and tailor treatment to the patient [38, 39, 45]. Educating patients about current knowledge of CRPS and the basis for the treatment approach seemed to increase participants’ willingness to engage in the treatment program [38]. This dynamic, time‐consuming treatment program may be challenging to apply in short‐term interventions and by health services with limited CRPS expertise [16, 45, 46].

*Regaining control* was the second key theme, aligning with the cure/control dimension of the CSM. This is a recurring theme reported in studies of CRPS and other chronic pain conditions [46, 47]. The participants expressed feelings of hopelessness and frustration from the pain and disabilities, lack of control and agency, and dependency on others. They faced difficulties finding the strength and motivation to regain agency. Several participants described a shift in focus from external factors, such as a pill or luck, toward a belief that they can take control. This corresponds to a change from external to internal locus of control beliefs [48]. Two factors facilitated this shift. The first, initial positive experiences from the treatment transformed their sense of helplessness into a belief that they are able to influence their situation and the CRPS. The second was the combination of illness comprehension and clinician support. Understanding the nature and mechanisms of the condition was a prerequisite for this shift in illness cognition, while motivation to adhere to the intensive training was fueled by the clinician’s continuous support and guidance.

Control was also a central theme in a study by Rodham et al. [46]. Their participants learned skills and techniques from healthcare professionals to self‐manage their CRPS, aiming to increase their sense of control and independence [46]. Limited access to expertise was emphasized as one of the key obstacles to maintaining control [46]. If any uncertainty arose about their home program or condition, they lacked sufficient help and support. This often led to noncompletion of exercises due to fear of doing something wrong and possibly worsening their condition. Not adhering to their program further increased their sense of hopelessness and loss of control [46]. A similar finding was reported in another CRPS study by Rodham et al. [49]. In both studies, a lack of access to expertise and support was a significant obstacle to maintaining control [46, 49]. In sum, an extensive follow‐up seemed necessary. In our study, participants were trained in self‐management and had frequent follow‐up consultations with the clinician. Our findings build on previous research and offer new perspectives on crucial factors in the outpatient treatment of CRPS, which requires intensive home‐based training [46].

The third theme was altered perceptions of the affected limb. The distorted perceptions of the affected limb, neglect, and avoidance reported by participants in our study are signs of BPD [20, 50]. This aligns with the literature where the majority (at least 72%) of patients with CRPS report BPD symptoms [51, 52]. Furthermore, BPD symptoms are more prevalent and severe in CRPS than in other chronic pain conditions [51, 53]. Our findings also concur with previous studies, demonstrating how these altered body perceptions were strong barriers to successful treatment and engagement of the affected limb in everyday life [3, 20, 54, 55]. Clearly, the BPD symptoms affected how the patients perceived the nature and implications of the condition, as well as how they related to it. Thus, the BPD affected the identity and consequence dimensions of the CSM. Lewis et al. (2012) reported a positive correlation between pain and BPD, where those with greater pain had more extensive BPD symptoms [56]. Although the direction of the relation is unclear, for example, whether BPD symptoms are driven by pain or vice versa [54, 56], efforts to reduce these symptoms are warranted and may potentially decrease the pain and improve the CRPS [54, 56]. Accordingly, GMI or mirror therapy alone is found to reduce BPD symptoms [54]. Furthermore, disease duration is a notable factor in CRPS, as longer disease duration is associated with greater body perception disturbance [56]. While disease duration varied substantially among the participants in our study, indications of BPD were reported by those with short as well as long disease duration. However, we cannot draw any conclusion on this issue based on the small sample size. Early focus on BPD and creating a more positive relationship with the affected limb may improve treatment engagement and help incorporate the CRPS limb in daily activities [56]. In light of these findings, it might benefit future treatment of CRPS to include BPD as an outcome measure [54, 56].

The patients perceived clinician support as crucial for treatment success. In several studies of CRPS, including ours, patients had prior experiences being met by a lack of trust and acknowledgment from the healthcare system [43, 45, 57]. CRPS is challenging to explain, and lack of comprehension may lead to a feeling of isolation and hopelessness [49, 58]. This, combined with the challenges of adhering to a demanding treatment program, can be hard to manage [46]. Our study highlights the importance of clinician support, and a novel finding is the emphasis by patients on being followed closely over time. Previous studies illustrate how unavailable expertise and support can hinder treatment adherence [38, 46]. Rodham and colleagues (2012) found that participants struggled to shift from an in‐hospital treatment program to self‐management at home [46]. In line with other studies, we found that access to CRPS expertise, providing guidance and support over time, is highly valued by the participants [45, 58, 59].

Despite recent advances in understanding CRPS, it remains a condition without a definite pathophysiological explanation, and patients may have personal ideas and beliefs about the condition and what it means to them [60]. This makes it challenging for physicians to explain and difficult for patients to grasp [45, 59].

In this study, the patients expressed a shift in their understanding of pain and CRPS. Before treatment, the patients perceived their pain as a threat that frightened them, leading to decreased usage of the affected limb. Illness comprehension, regaining control, reintegrating the affected limb, and strong clinician support seemed vital to the positive change. Given GMI’s high demands on training motivation [15], our findings enlighten some critical factors for treatment adherence and outcome. Adherence to an individually tailored treatment by an experienced clinician seems essential for a successful treatment [38, 46].

### 4.1. Limitations

Due to the study’s methodology and the limited sample size of ten participants, our findings may not be easily generalizable to the broader CRPS population. The decision to use a qualitative approach was driven by the goal of capturing the depth and complexity of patients′ experiences, acknowledging that the insights obtained are context‐specific. Instead of traditional generalizability, the qualitative approach emphasizes transferability, understood as the applicability of the findings to similar contexts. We made efforts to enhance transferability by providing rich empirical data supporting the findings. Potential recall bias is a common limitation in retrospective interview studies. While we cannot eliminate recall bias regarding patients’ experiences, we minimized the risk by selecting participants who were currently undergoing or had recently undergone the treatment.

Additionally, with nine out of the ten participants being female, including more male participants might have provided valuable insights into a spectrum of male experiences and potential gender differences in patient experiences. Furthermore, in this exploratory qualitative study, patient experiences may reflect both general aspects of participating in an intensive rehabilitation program as well as the specific techniques of GMI and TD treatment provided in this study. The co‐author and physician LD made efforts through reflexive practice to avoid crossing the lines between the roles of a researcher and a CRPS physician [36].

## 5. Conclusion

This study provides insight into the experiences and challenges faced by patients with CRPS who participated in an intensive outpatient GMI and TD program. The participants emphasized illness and treatment comprehension, regaining control, altered body perceptions, and clinician support as critical topics. The findings may aid future clinicians in tailoring treatment programs to individual patients, thereby increasing the likelihood of adherence and successful outcomes. Further research is needed on larger samples to establish how these factors relate to one another, identify predictors of adherence and outcomes, and determine how they may be incorporated into clinical practice.

## Author Contributions

Lena Danielsson, Gunnvald Kvarstein, and Svein Bergvik designed the clinical trial. Isak Furu Krogstad, Lena Danielsson, Gunnvald Kvarstein, and Svein Bergvik designed the interview study. Isak Furu Krogstad administered the recruitment of participants and conducted the interviews. Isak Furu Krogstad, Louisa Palmi Danielsson, and Svein Bergvik analyzed the data. Isak Furu Krogstad drafted the manuscript.All authors contributed in preparation of the manuscript. Svein Bergvik submitted the manuscript.

## Funding

No funding was received for this manuscript.

## Disclosure

All authors revised it and provided final approval.

## Conflicts of Interest

The authors declare no conflicts of interest.

## Data Availability

In accordance with the Norwegian Health Research Act and the approval of the Regional Committee for Medical and Health Research Ethics, data from this patient interview study are not permitted to be publicly available.
